# The contemporary role of surgery in kidney cancer

**DOI:** 10.3747/co.v16i0.410

**Published:** 2009-05

**Authors:** J.B. Lattouf, Q.D. Trinh, F. Saad

**Keywords:** Kidney cancer, surgery, laparoscopy, minimally invasive technique

## Abstract

The diagnosis and treatment of renal cell carcinoma (rcc) has been the subject of major changes since the late 1980s. Initially, surgery was the only treatment available, but more recently, systemic therapies have been developed, and their introduction has modified some of the surgical indications for rcc. In addition, refinements in surgical technique and the introduction of minimally invasive approaches have revolutionized patient care and bear the promise of even more improvements to come. This paper provides an up-to-date overview of recent developments in the surgical treatment of rcc.

## 1. INTRODUCTION

Until recently, renal cell carcinoma (rcc) was considered mainly a surgical disease, because it is, by and large, one of the most chemo- and radioresistant tumours. Since the first nephrectomy was performed for neoplastic disease by Langenbuch in 1877 1, huge strides have been made in refining the surgical indications and technical aspects of surgery for rcc. During the quarter century since the mid-1980s, new imaging modalities such as ultrasonography, computed tomography (ct), and magnetic resonance imaging (mri) have come into widespread use. These diagnostic tests now allow for better preoperative staging and surgical planning of interventions. On the other hand, during that same period, imaging has also shifted the stage at which rcc is being diagnosed and treated, and now a question arises: Is surgical treatment still indicated for very small renal lesions 2–4, which in many cases are turning out to have an indolent natural history? At the other end of the spectrum, patients suffering from advanced-stage rcc have benefited from the introduction of systemic immune therapy, with some having complete and durable responses 5. Surgical treatment retains its place in this setting in the form of cytoreductive nephrectomy 6,7. Further, the discovery of the von Hippel–Lindau (*VHL*) gene and the understanding gained of the molecular biology of the disease have set the stage for an exciting new era of targeted therapies 8. However, the role of cytoreductive nephrectomy with the new tyrosine kinase inhibitors (tkis) remains to be defined.

The present review touches on the indications and controversies surrounding surgical treatments for rcc. The evolution of surgical techniques and the introduction of laparoscopy are also discussed.

## 2. SURGICAL TREATMENT FOR LOCALIZED RCC

### 2.1 Radical Nephrectomy

In 1963, Robson and colleagues published the seminal work that laid the foundations of surgical treatment for rcc as we know it today 9,10. Key surgical steps in achieving adequate and safe oncologic control were identified, including early vascular control and renal excision preserving Gerota’s fascia, thus ensuring that surgical margins are away from the tumour. Other concepts put forward by Robson included adrenal gland excision within Gerota’s fascia and extended ipsilateral lymphadenectomy from the crus of the diaphragm to the bifurcation of the aorta. These latter practices are not universally applied in the contemporary surgical approach, mostly because of a stage shift in rcc toward localized disease and because of the new imaging modalities. Nonetheless, the description of a radical nephrectomy remains one of the pillars of the treatment of localized rcc; all technical refinements developed thereafter have been guided by its basic principles.

Contemporary treatment of rcc is a factor of tumour size and location, presence of venous thrombus, and patient characteristics. Many incisions have been described, including flank, subcostal, midline, and thoraco-abdominal. Long-term oncologic results for open radical nephrectomy have been consistently reproduced and serve as a benchmark for other surgical modalities. Although results from the initial series by Robson *et al.* showed a 5-year survival of 66% in localized disease 10, outcomes improved significantly in later studies. For patients with stage i disease, 5-year survival in contemporary series is now established at 74%–95% 11–15. Table i shows contemporary 5- and 10-year survival data after radical nephrectomy.

#### 2.1.1 Role of Adrenalectomy

The incidence of adrenal metastasis from rcc in contemporary series has been reported at 2.7%–5.7% 16–19. This low occurrence, coupled with modern imaging modalities, has led to many questions about the role of systematic adrenalectomy during radical nephrectomy as initially suggested by Robson. Motivations for adrenal sparing include the possibility of relapse in the contralateral adrenal, the potential for hormone imbalances, and the desire to achieve shorter operative times. Indications for adrenalectomy vary according to author and include large tumours, upper pole tumours, and adrenal anomalies noted on preoperative imaging or intraoperatively 20.

In a large contemporary European series of 1179 nephrectomies, Antonelli *et al.* found no adrenal metastases associated with tumours under 4cm. Tumour location within the kidney was not predictive of adrenal involvement, and imaging by ct had a low sensitivity at 47%. Thus, the authors suggested the ipsilateral adrenal be spared only for tumours smaller than 4cm, independent of tumour location, provided that preoperative imaging of the adrenal gland is normal 18. Another study by Siemer *et al.*, also with a large sample (*n* = 1010), arrived at the same conclusions, underlining the importance of the 4-cm cut-off and the irrelevance of tumour location 21. It therefore seems prudent to consider clinical stage as the sole indication for adrenalectomy when radical nephrectomy is considered.

#### 2.1.2 Role of Lymphadenectomy

Until recently, the role of lymphadenectomy in rcc surgery was a subject of controversy. Although the incidence of lymph node metastases as initially published by Robson *et al.* was 30%, modern series report occult metastases in only 3.3% of cases 10,22. However, even if lymphadenectomy may provide for accurate staging with no increased morbidity, its benefit with respect to survival and disease progression has not been established. A retrospective study of rcc surgical patients in the U.S. Surveillance, Epidemiology, and End Results database between 1983 and 1998 showed no survival advantage when extensive lymphadenectomy was performed in localized disease 23. Furthermore, the published results of the randomized phase iii European Organization for Research and Treatment of Cancer (eortc) 30881 trial assessing radical nephrectomy with and without lymphadenectomy confirm the lack of a survival difference associated with the added lymph node dissection 24. However, the value of lymphadenectomy in clinically node-positive patients undergoing cytoreductive nephrectomy for advanced disease remains to be clarified 25.

#### 2.1.3 Surgery with Venous Thrombus

One particular challenge in rcc surgery is macroscopic tumoural extension into the venous system, namely the vena cava, which occurs in 5%–10% of cases 26. When rcc is still localized in such a setting, surgery remains the mainstay of treatment 27,28. The largest series published to date on venous tumoural extension relates a multicentric experience from Europe, regrouping 1192 patients operated on between 1982 and 2003 29. The authors reported median survivals of 52 months for thrombi limited to the renal vein, 25.8 months for thrombi in the subdiaphragmatic vena cava, and 18 months for thrombi above the vena cava. A difference in survival was detected between tumours in the renal vein and those in the vena cava. However, the level of the tumour thrombus in the vena cava did not significantly affect survival.

The foregoing findings are very similar to results from the largest single-centre experience published to date from the Mayo clinic, where the 5-year survival was 49.1% for tumours limited to the renal vein and 26.3%–39.4% for tumours in the vena cava 30. This last study—and others—also found statistically different survivals for renal vein as compared with vena cava thrombi, but the level of the thrombus in the vena cava was not predictive of survival 31. It seems, in fact, that biologic aggressiveness in such settings is dictated mainly by tumour grade and not by thrombus extension 32. Technical challenges are greater for thrombi extending above the diaphragm, because these necessitate cardiopulmonary bypass and a team approach. However, surgical refinements, including hepatic transplantation techniques and preoperative renal artery embolization, may help to circumvent this problem 33,34.

#### 2.1.4 Laparoscopic Radical Nephrectomy

Laparoscopic radical nephrectomy was first described for neoplastic disease by Clayman *et al.* 35 in 1991. The many interesting advantages, including a lesser postoperative analgesia requirement, shorter hospital stay, quicker convalescence, and improved cosmesis, have fuelled enthusiasm for this new approach and made it the preferred modality for localized rcc. Oncologic outcomes have been published with medium- and long-term follow-up 36–41 (Table ii), and comparative studies show equivalence of laparoscopic radical nephrectomy and the open approach 43–45.

Laparoscopy has been applied successfully to renal tumours as large as 16 cm 46—albeit follow-up for this patient population is still limited, and few publications are available. Furthermore, laparoscopic radical nephrectomy is feasible in the overweight patient, and although technically more challenging, this approach may result in superior perioperative outcomes as compared with the open technique 47. Finally, centres of expertise have reported laparoscopic radical nephrectomies with renal vein and vena cava tumour thrombi, confirming the safety and feasibility of the technique 48–50. In the view of many experts in the field, laparoscopic radical nephrectomy should be the standard of care for localized rcc whenever technically feasible and when a partial nephrectomy is not indicated.

Access to the kidney may be obtained in a trans- or retroperitoneal fashion, with each route having its advantages and drawbacks. The retroperitoneal approach provides faster access to the renal hilum and may be the best choice for patients with posterior tumours or with multiple prior abdominal surgeries. The transperitoneal approach remains the most popular, however, because it affords a larger working space and more familiar anatomic landmarks. Notwithstanding theses differences, both approaches seem to be equivalent in terms of perioperative outcome 51.

To ease the learning curve, hand-assisted laparoscopic radical nephrectomy (halrn) was developed in parallel with the pure laparoscopic approach. It consists of taking advantage of the incision from which the specimen is to be extracted to position a hand-assist device that allows for the intra-abdominal introduction of the operator’s hand. This technique offers the added advantage of tactile feedback and allows for manual compression when brisk bleeding is encountered. Although pure laparoscopic nephrectomy, as compared with halrn, may show a trend toward fewer complications in high-risk patients (American Society of Anesthesiologists score ≥3) 52, the halrn approach was shown to be equivalent to open radical nephrectomy on long-term follow-up 53.

### 2.2 Partial Nephrectomy

Initially, the indications for partial nephrectomy in malignant disease were limited to imperative conditions, such as renal insufficiency, a solitary kidney, familial renal tumours, or underlying conditions that could jeopardize the contralateral renal unit. However, since the advent of ct and magnetic resonance imaging, a significant stage shift has occurred toward incidentally discovered smaller and localized renal lesions 4,54,55. In contrast with an overall rate of malignancy of 90%, these smaller tumours (<4 cm) tend to have a benign histology rate of up to 23% 56,57. Additionally, although nephrectomy in the younger population may not result in long-term functional impairment of global renal function 58, it has been shown that radical nephrectomy in the rcc population may increase the lifetime risk for dialysis because of previous underlying medical conditions 59–61. These considerations, compounded by a risk of contralateral metachronous tumour recurrence of up to 4.25% 62,63, argue in favour of a nephron-sparing surgery (nss) approach, even in patients with normal contralateral renal units.

The goal of nss is to resect the tumour with negative margins, while preserving the largest possible amount of normal parenchyma. This procedure may involve a more complex set of technical steps than does radical nephrectomy, including preservation of the renal pedicle during hilar dissection, the need for kidney cooling during prolonged renal ischemia, and finally the need for a complex collecting system and vascular repair after tumoural resection. In some cases, the use of intraoperative ultrasound may also be required to delineate tumour location or the relationship of the tumour to major intrarenal vessels. Intraoperative frozen sections to rule out positive margins were advocated initially, but have not been universally adopted 64.

#### 2.2.1 Open NSS

Studies on open nss with a normal contralateral kidney have shown that, for tumours under 4cm in size, oncologic control in terms of 5- and 10-year survival is equivalent to that seen with radical nephrectomy 65–68. Partial nephrectomy has been attempted for larger tumours, but follow-up of these patients in the literature is scarce 69–72. Overall, nss is not associated with more complications than is radical nephrectomy 73–75. It is thus widely accepted that partial nephrectomy constitutes the “gold standard” for the treatment of localized rcc smaller than 4 cm.

The eortc 30904 randomized controlled study, whose objective was to compare nss with radical nephrectomy, has been completed. The results with regard to oncologic outcomes are awaited with great anticipation 76.

#### 2.2.2 Laparoscopic NSS

Because of the complex technical steps involved in partial nephrectomy, the laparoscopic approach to this procedure was slow to emerge as a viable alternative. One of the main initial hurdles to overcome was hemostasis after parenchymal incision, and initial laparoscopic nss procedures were performed mainly for small exophytic renal lesions with very little parenchymal involvement. With evolving experience, intracorporeal knot-tying and hilar clamping emulating the open approach came into use, and the treatment of intraparenchymal lesions became feasible 77. Because of prolonged warm ischemia times at first, novel and innovative methods of cold ischemia have been described with varying degrees of success and varying advantages and caveats. These methods have included ice-slush cooling, intra-arterial cold saline perfusion, and collecting system cold perfusion using a ureteral catheter 78–81. However, renal cooling is less frequently used now, because clamping times have been significantly reduced with evolving experience.

Medium-term follow-up of oncologic outcomes with laparoscopic nss for small renal tumours is becoming available 82,83. The data reveal excellent results that are comparable to those achieved with the open approach. Increasingly larger and more complex tumours are being managed using laparoscopic nss 84,85. However, because of the complexity of the procedure and the paucity of long-term follow-up data, laparoscopic nss remains an approach under investigation that should be performed only in centres with the requisite experience.

#### 2.2.3 Robotic NSS

Tele-robotic surgical systems have recently been introduced with the main goal of reducing the complexity of intracorporeal suturing, often at difficult angles, as compared with conventional laparoscopy. Gettman *et al.* first described robot-assisted laparoscopic partial nephrectomy (ralpn) in 2004 86. In the initial report of 13 cases by these authors, mean lesion size was 3.5cm (range: 2.0–6.0cm), mean operative time was 215 minutes (range: 130–262 minutes), and mean blood loss was 170mL (range: 50–300mL). Positive margins were reported in 1 case (7.7%). The conclusion was that ralpn is technically feasible and safe. One concern was the potential for delayed intervention in situations of urgent conversion such as massive hemorrhage. Nevertheless, other groups have reported the feasibility and safety of ralpn 87–93. Interestingly, Aron *et al.* published a matched-pair series that compared the experience between robotic and laparoscopic partial nephrectomy at the Cleveland Clinic 91. Overall, no differences were observed in perioperative variables (warm ischemia time, estimated blood loss, surgery time, length of stay). Fewer ports were used during laparoscopy. Renal functional outcomes, transfusion rate, and complication rates were comparable. Two ralpn cases required conversion to standard laparoscopy. The authors concluded that ralpn is a developing procedure and that further experience is necessary to determine the relative merits of ralpn. At the date of writing, oncologic outcomes are yet to be determined, but ralpn is not likely to improve surgical and oncologic outcomes over laparoscopy. It might, however, permit more urologists to perform this complex procedure, allowing it to evolve into mainstream practice.

## 3. SURGICAL TREATMENT FOR METASTATIC RCC

### 3.1 Cytoreductive Nephrectomy

The rationale of cytoreductive nephrectomy in metastatic rcc is twofold: first, to remove a large tumour burden that could theoretically be immunosuppressive, and second, to prevent potential local complications in the course of systemic therapy. Systemic therapy for rcc has evolved over the past 20 years from immune-based agents such as interferon alfa and interleukin-2, to the most recent small-molecule inhibitors, including sorafenib, sunitinib, and temsirolimus. In the setting of systemic immunotherapy, cytoreductive nephrectomy may be performed before treatment and has been shown to prolong survival in Southwest Oncology Group (swog) and eortc trials using interferon alfa 6,7,94. However, nephrectomy before immunotherapy has the caveat, in up to 60% of patients, of possible complications that may preclude these patients from ever receiving immunotherapy 95–97. Some investigators have therefore sought to assess systemic therapy as first-line therapy so as to delineate responders, considering them to be the only candidates likely to benefit from subsequent surgery. Using this paradigm, the 31%–50% of patients who are non-responders are spared the morbidity of nephrectomy 98,99.

The laparoscopic approach to cytoreductive nephrectomy offers the advantage of reduced recovery time and perhaps the opportunity to initiate systemic therapy earlier than might occur with the open approach. Laparoscopic nephrectomy has been assessed, and Walthers *et al.* from the National Cancer Institute published a series in which the technique was shown to be safe and feasible 100.

In the era of tkis, the role of cytoreductive nephrectomy is not clearly defined. A discussion of the various trials pertaining to tki therapy for rcc is beyond the scope of this review, and yet it should be emphasized that most patients assigned to treatment with a tki in randomized trials had already undergone nephrectomy. Thus, to remain comparable with the experimental setup, it seems reasonable to proceed to surgical cytoreduction in properly selected patients in the clinical setting.

### 3.2 Metastasectomy

Few studies are available on the effects of metastasectomy, whether used alone or in combination with systemic therapy 101–103. It is commonly accepted that a better prognosis follows from resection of lung metastases than from extrapulmonary lesions. Patients with synchronous metastases fare worse than do those with metachronous metastases. Furthermore, the longer the time from primary tumour resection to metastasectomy, the better the chances of achieving cure. The number of resectable metastases and the number of re-resections do not seem to affect the overall prognosis. Finally, a resection with the goal of achieving curative oncologic control results in far better survival than does palliative resection only. A retrospective study from the MD Anderson Cancer Center showed that, when it comes to exclusively local recurrence, surgery plus systemic therapy portends a 5-year survival of 50%, which is significantly superior to the survival seen with surgical resection alone 104.

## 4. CONCLUSIONS

The surgical treatment of rcc has evolved tremendously since the end of the 1980s. It remains the most efficient modality to cure localized disease, and it is the “gold standard” by which newer, minimally invasive therapies should be assessed. With the advent of new systemic therapies for rcc, many questions have been raised that remain to be answered concerning the role and timing of surgical cytoreduction. As with all other fields in oncology, the future of rcc surgery lies in identifying the patients that will eventually benefit the most from surgical treatment and in sparing those that will not. The ultimate answers will probably come from the discovery of novel biomarkers, resulting in genetically tailored treatment for each individual patient.

## Figures and Tables

**TABLE I:** Studies of 5- and 10-year survival in radical nephrectomy for renal cell carcinoma according to stage (i to iv)

*Reference*	*Survival (%)*							
	*5-Year*				*10-Year*			
	*i*	*ii*	*iii*	*iv*	*i*	*ii*	*iii*	*iv*
Kinouchi *et al.*, 1999^11^	99	92	70	24	93	73	49	19
Guinan *et al.*, 1995^12^	65	51	—	—	—	—	—	—
Javidan *et al.*, 1999^13^	95	88	59	20	96	92	43	14
Tsui *et al.*, 2000^14^	83	57	42	28	—	—	—	—
Gettman *et al.*, 2001^15^	99	90	66	39	97	84	53	32

**TABLE II:** Laparoscopic radical nephrectomy for T1–2 disease: overall survival with medium- and long-term follow-up

*Reference*	*Survival [cancer-specific/overall (%)]*	
	*5-Year*	*10-Year*
Chan *et al.*, 2001^37^	85/—	—
Hemal *et al.*, 2007^38^	88/85.6	—
Ono *et al.*, 1999^39^	95/—	—
Permpongkosol *et al.*, 2005^40^	97/85	97/76
Saika *et al.*, 2003^41^	94	—
Portis *et al.*, 2002^42^	98/81	—
